# Mining Public Metagenomes for Environmental Surveillance of Parasites: A Proof of Principle

**DOI:** 10.3389/fmicb.2021.622356

**Published:** 2021-06-30

**Authors:** Frits F. J. Franssen, Ingmar Janse, Dennis Janssen, Simone M. Caccio, Paolo Vatta, Joke W. B. van der Giessen, Mark W. J. van Passel

**Affiliations:** ^1^Centre for Zoonoses and Environmental Microbiology, National Institute for Public Health and the Environment, Bilthoven, Netherlands; ^2^Department of Infectious Diseases, Istituto Superiore di Sanità, Viale Elena Regina, Rome, Italy

**Keywords:** metagenome analyses, parasite detection, signature sequences, *Cryptosporidium parvum*, environmental metagenomes

## Abstract

Parasites often have complex developmental cycles that account for their presence in a variety of difficult-to-analyze matrices, including feces, water, soil, and food. Detection of parasites in these matrices still involves laborious methods. Untargeted sequencing of nucleic acids extracted from those matrices in metagenomic projects may represent an attractive alternative method for unbiased detection of these pathogens. Here, we show how publicly available metagenomic datasets can be mined to detect parasite specific sequences, and generate data useful for environmental surveillance. We use the protozoan parasite *Cryptosporidium parvum* as a test organism, and show that detection is influenced by the reference sequence chosen. Indeed, the use of the whole genome yields high sensitivity but low specificity, whereas specificity is improved through the use of signature sequences. In conclusion, querying metagenomic datasets for parasites is feasible and relevant, but requires optimization and validation. Nevertheless, this approach provides access to the large, and rapidly increasing, number of datasets from metagenomic and meta-transcriptomic studies, allowing unlocking hitherto idle signals of parasites in our environments.

## Introduction

Parasites are eukaryotic pathogens, broadly divided into single cell (protozoa) and multicellular organisms (nematodes, cestodes, and trematodes), which cause infection and disease in vertebrate hosts. Parasites often have complex developmental cycles and are transmitted via direct contact, food, vectors or the environment. The latter through the uptake of environmentally persistent parasite stages (eggs, larvae or (oo)cysts) that contaminate e.g. water, soil or, food (e.g., fresh produce) (Chalmers et al., 2020).

Most parasites cannot be cultured using *in vitro* systems and detection procedures rely on microscopy, alone or in combination with immunological or histochemical techniques, and/or molecular methods (e.g., PCR and sequencing, qPCR). Jointly, these tools allow the detection of parasites in different biological samples as well as food matrices and environmental samples (Amoah et al., 2017; Sengupta et al., 2019; Chalmers et al., 2020). However, difficult-to-analyze matrices (e.g., water, soil, feces, sludge, food) still require laborious concentration and purification steps (Skotarczak, 2009; Sroka et al., 2013; Amoah et al., 2017; Sengupta et al., 2019). Therefore, metagenomic sequencing may offer an attractive method for unbiased parasite detection.

Pallen (2014) introduced the term diagnostic metagenomics, which was defined as the detection and characterization of pathogens from untargeted (shotgun) sequencing metagenomic data (Pallen, 2014). Diagnostic metagenomic studies predominantly focused on the detection, typing and further characterization of bacteria and viruses, with sample collection and preparation optimized for these microbiological agents. In such studies, the extraction and amplification of DNA or RNA is often followed by downstream removal of “contaminating” eukaryotic (host) sequences. Additionally, tools developed for taxonomic classification of metagenomic reads such as Kraken (Wood and Salzberg, 2014; Wood et al., 2019), VirFinder (Ren et al., 2017), and Metaxa2 (Bengtsson-Palme et al., 2015) are particularly suitable for the analysis of prokaryotes and viruses. To improve the detection of parasites in metagenomic experiments, Wylezich et al. (2018) optimized NGS procedures for simultaneous DNA and RNA isolation from bacteria, viruses and parasites, from various matrices such as liquids, tissues, feces, as well as processed and non-processed foods (Wylezich et al., 2018).

Phylogenetic classification and/or species confirmation of metagenomic reads depends on successful mapping of these reads against reference sequences. Several public databases such as NCBI,^1^ EupathDB,^2^ and Wormbase^3^ provide comprehensive whole genome reference information of protozoan and helminth parasites.

Eukaryotes are increasingly also being surveyed in metagenomic datasets, e.g., exploration of fungi in public animal metagenomes (Donovan et al., 2018). Despite the fact that endogenous parasites are often regarded as contaminants of animal sequence assemblies (Borner and Burmester, 2017), such “contaminants” provide valuable information for parasitologists (Lopes et al., 2017). One example is provided by Beghini et al. (2017), who evaluated human gut metagenomes for the presence of *Blastocystis* DNA, and used these metagenomic datasets for both parasite epidemiology and full genome reconstruction (Beghini et al., 2017). Wylezich et al. (2019) used rRNA sequence-based metagenome analysis to demonstrate various protozoan parasites in pig feces (Wylezich et al., 2019).

The protozoan parasite *Cryptosporidium parvum*, a major cause of gastroenteritis in humans and animals worldwide, is a well-known example of a food- and water-borne pathogen. The infective stage of the parasite (the oocyst) is shed with the host feces that can contaminate the environment. Therefore, DNA of this parasite could be present in a range of environments, which could be investigated by querying metagenomes originating from these environments.

The aim of the current study was to provide a proof of principle for the detection of parasites by mining metagenomic public data bases, which may be used for their environmental surveillance. Moreover, the specificity of this method was evaluated, based on the idea that identification of parasites in this type of data requires highly specific and reliable results.

## Data Processing

**FIGURE 1:**
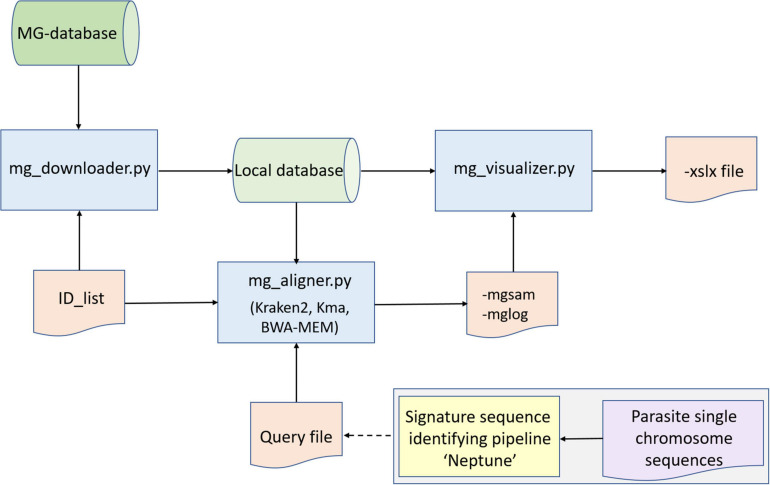
Conceptual layout of the metagenome pipeline to detect parasites. Basically, the pipeline consists of three Python scripts (blue boxes) that receive input from databases (green cylinders) and two input files (ID_list and Query file), leading to output files mgsam, mglog and xslx file. Metagenome database sequence reads are checked for completeness of sequence- and metadata by Python script mg_downloader.py. Sequence reads in the database are classified to species level with mg_aligner.py. This may take place using Kraken-2, which gives an overview of what species may be present in an environment, or alternatively, reference sequences (orange box) may be used to mine the database for specific species, using e.g. K-mer Aligner (Kma) or Burrows-Weeler Aligner (BWA-MEM).Optionally, an independent pipeline may be used to identify specific (signature) sequences (yellow box), which can be used as reference sequence (Query file, orange box). During post-processing, confirmation of retrieved reads may take place (e.g., by BLASTn). All results are merged into two reports: mgsam and mglog which are processed to an xlsx file with mg_visualizer.py.

We set out to develop a proof of principle bio-informatics pipeline to query public environmental metagenomes for parasite specific sequences (see Figure 1 for a conceptual outline of the pipeline and Supplementary File 1). The pipeline was deployed locally on our Institute’s high performance cluster and the time to run a search varied from overnight (five metagenome project numbers and specific query sequences) to several days.

CPU time and total computing time for Kraken2 and BLASTn in comparison to taxonomic classification tools MMseq2 (Mirdita et al., 2020) and Sourmash (Ondov et al., 2016) was determined, in order to evaluate scalability of our pipeline (Supplementary File 4 and Supplementary Figures 3, 4). The full Python pipeline code is available upon reasonable request.

To provide a global inventory of the eukaryote species present in an environment, classification tools are needed to assign reads to a taxonomic level, preferably species level. In this study, we used two approaches for this purpose. Firstly, we used Kraken, and its improved version Kraken-2 (Wood and Salzberg, 2014; Wood et al., 2019), which uses exact *k*-mer matches (short sequences of around 32–35 nucleotides) and allows for a fast evaluation and taxonomic identification of reads to the lowest common ancestor. Secondly, we used the Burrows-Wheeler Aligner (BWA-MEM; Heng, 2013), or the k-mer alignment (KMA; Clausen et al., 2018), to identify taxa of interest based on the alignment of metagenomic reads to selected query reference sequences. The parameter for parasite taxon assignment was identification of the lowest common ancestor for Kraken 2, based on a confidence score of 0.05, and 100% alignment identity for Kma and BWA-MEM.

The feasibility to use metagenome analysis to evaluate the presence of parasites in different matrices relies on presence and correct interpretation of metadata. e.g., environmental source types such as “water” may be further subdivided into fresh water, sea water, thermal spring, or coastal water. Another example is “sediment,” which may include subtypes like marine sediments, Alpine glacier sediments or river sediments. Often, the description of a metagenomic project contains the required information, yet not uniformly provided, which may obstruct correct automatic extraction of this information. FAIR guiding principles have been set up for data management and stewardship (Wilkinson et al., 2016), but compliance could still be improved. The amount of data in public metagenomic databases is rapidly expanding, which may force users to decide which data to retrieve and store at forehand, due to local storage and computing limitations. Therefore, criteria should be carefully formulated for inclusion or selection of data and data quality (e.g., deletion of short reads and incomplete metadata).

## Metagenome Analysis of Parasites

Confident detection of sequence signals of a particular organism in a metagenomic dataset depends on the match between metagenomic reads and the query sequence, in combination with the specificity of the query sequence(s) used for species identification. Ideally, the nucleotide identity between metagenomic reads and the query sequence, over an acceptable sequence length, is 100%.

We started by using the small subunit ribosomal DNA (18S rDNA) sequence to query metagenome data available at the MG-RAST database, and we choose an example of a parasite expected to occur in one environment only, namely *Entamoeba gingivalis* in the human oral cavity. The MG-RAST database (MG-RAST, downloaded September 2019, 5557 metagenomes out of 29,903 approved, each containing >999 sequences with an average sequence length >84 nucleotides and corresponding metadata) was queried (general settings: coverage >80%, length >75 nucleotides (nt), meta check “True,” *P*-value <0.05) with the 18S rDNA sequence of a parasite species expected to occur in one environment only: *E. gingivalis* in the human oral cavity. Eleven metagenomic reads of 52–241 nt matched at 100% coverage and 100% identity to *E. gingivalis* 18S rRNA; 9 of these reads from the human oral environment were subsequently confirmed as *E. gingivalis*, using NCBI BLASTn. At 98–99% nucleotide identity, which still may be regarded as very high, reads that were retrieved as *E. gingivalis* were in majority confirmed by BLASTn as belonging to the closely related species *Entamoeba suis* (25 reads, 100% match, see Supplementary Figure 1).

To further evaluate the use of 18S rDNA sequence, we expanded the study to additional parasites including *Balantidium coli*, *Cryptosporidium hominis*, *C. parvum*, *Entamoeba coli*, *E. gingivalis*, *Trichomonas tenax*, *Trichomonas vaginalis*, and *Tritrichomonas foetus*.

Some of these parasites are expected to only occur in a single environment (e.g., *E. gingivalis*, as shown above), whereas others may be present in a range of environments (*B. coli*, *Cryptosporidium* spp., *E. coli*, *T. vaginalis*, and *T. foetus*). Firstly, we retrieved 18S rDNA gene sequences of the selected parasites to query a locally installed MG-RAST database as described above. Retrieved reads were aligned using the Burroughs-Wheeler aligner (BWA; Li and Durbin, 2009) and matching reads were confirmed using NCBI BLASTn. Most aligned reads were retrieved from metagenomes of environments in which the parasite was expected to occur (see Supplementary Table 1). The absence of reads from expected environments is not surprising, considering that parasite abundance may be very low (e.g., *Cryptosporidium* spp. in wastewater) and that some environments are vastly underrepresented in metagenomic databases (e.g., *T. vaginalis* from the female reproductive tract or *T. foetus* from the bovine or feline reproductive tract). In the case of the 18S rDNA sequence from *Cryptosporidium* spp., over 1,500 reads from different environments aligned with the reference sequence, but only four reads were confirmed to be specific for *C. parvum*; these reads were from a calf metagenome study (MG-RAST project numbers 4537110.3, 4536848.3, 4536849.3, and 4537108.3). All other reads were specific only at the genus level (or higher) and BLASTn verification yielded non-specific results, spanning fungi, yeasts, other protozoa, and algae (data not shown).

Therefore, the use of 18S rDNA sequences to query metagenome databases was considered not specific enough, since many reads matched regions highly conserved among different species, preventing identification of the target organism.

To expand evidence of parasite DNA sequences in metagenomic samples obtained by using the 18S rDNA as reference, the MG-RAST project numbers that were positive for *Cryptosporidium* from wastewater/sludge and those from host-associated environment, were queried again with the same settings as described above, but this time using the whole genome sequence of *C. parvum* Iowa II strain as reference. Reads from two environments matched the reference genome, namely water (MG-RAST project number 4622705.3) and bodily fluid of calves (MG-RAST project numbers 4537110.3, 4536848.3, 4536849.3 and 4537108.3) (43,802 reads in total).

These metagenomic projects were queried again using the whole *C. parvum* chromosome 6 sequence as a reference; 0.03–2.16% of total reads were assigned to *Cryptosporidium* (Table 1A). For the vast majority of these assigned reads, *C. parvum* was confirmed using KMA and subsequently by BLASTn at high confidence in all bodily fluid samples, but not in water (Table 1B). Still, a minority of reads was assigned to *C. hominis* and *Cryptosporidium ubiquitum*, although at lower confidence (Table 1B and Supplementary File 2).

**TABLE 1:** Querying the MG-RAST database with chromosome 6 sequence of *Cryptosporidium parvum* Iowa II strain yielded two environments and five project numbers.

**A.** Metagenome project description and analysis.								
**Project nr.**	**Continent**	**Country, Location**	**Environment**	**Total nt count**	**Reads count**	**% Reads**	**Assigned reads**	**Assigned% reads**

4622705.3	South America	Brazil, Sao Paulo, Brazil	Fresh water	26,668,719	13,612	0.05	7,055	0.03
4537110.3	North America	Canada, Edmonton	Calf mid jejunum	167,401	402	0.24	267	0.16
4536848.3	North America	Canada, Edmonton	Calf distal jejunum	199,721	1,399	0.70	977	0.49
4536849.3	North America	Canada, Edmonton	Calf ileum	104,749	1,912	1.83	1,398	1.34
4537108.3	North America	Canada, Edmonton	Calf distal jejunum	147,855	4,382	2.92	3,199	2.16

**B.** Blast confirmed reads count and median E-value (range) per *Cryptosporidium* species.							

**Project nr.**	**Count**	***C. parvum* (taxon id 5807 & 353152)**	**Count**	***C. hominis* (taxon id 353151)**	**Count**	***C. ubiquitum* (taxon id 857276)**	**Total counts**

4622705.3	0	–	1	5.40E-08	0	–	1
4537110.3	255	6.04E-81 (9.62E-92–4.36E-81)	9	6.48E-75 (8.37E-86–2.20E-53)	2	– (5.58E-63–2.24E-48)	266
4536848.3	944	6.04E-81 (9.62E-92–5.47E-40)	24	3.92E-73 (3.33E-91–1.73E-40)	7	9.16E-47 (2.48E-67–9.12E-20)	975
4536849.3	1,351	1.45E-82 (9.62E-92–7.75E-42)	32	1.18E-73 (1.16E-90–3.97E-50)	15	7.20E-42 (5.40E-63–2.89E-20)	1,398
4537108.3	3,095	4.19E-83 (9.62E-92–4.12E-39)	84	2.42E-77 (9.62E-92–3.79E-42)	19	3.52E-46 (4.23E-77–1.35E-23)	3,198

*A. In two environments, fresh water used for irrigation and calf bodily fluids, 0.05–2.16% of reads showed homology with the *Cryptosporidium* query, of which 0.03–2.16% reads were assigned, using BWA-MEM. B. Of these assigned reads, three *Cryptosporidium* species could be confirmed with NCBI BLASTn in all four calf bodily fluid projects. Total count and median E-values are provided per *Cryptosporidium* species and minimum – maximum range is given between brackets. Per *Cryptosporidium* species the column ‘Total counts’ relates to total number of confirmed reads per project number. In the water project 4622705.3 only one single read could be confirmed.*

In the water metagenome (MG-RAST project number 4622705.3), other eukaryotic organisms, such as fungi, yeast and six protozoan parasites other than *C. parvum* were identified by BLASTn confirmation, which means that the DNA sequences to which reads matched were highly unspecific (data not shown).

Table 2 shows in detail for MG project number 4537108.3 species to which the reads were mapped by KMA (see also Supplementary File 2 and Table 2). Although the vast majority of reads was confirmed as *C. parvum* at low median E-values (thus high confidence) by BLASTn, taxon classification was unequivocal. This shows that the reference sequence that had been used was still not specific enough to determine whether *C. parvum* is present in a given environment at high confidence, without the need for confirmation.

**TABLE 2:** Reads mapped to different *Cryptosporidium* species of MG project number 4537108.3 showed variable specificity.

**Species**	**Taxon ID**	**Median E-value**	**E-value range**	**n**
**Reads mapped to *Cryptosporidium***				
*C.parvum*	5807/353152	4.19E-83	9.62E-92–4.34E-48	153
*C.hominis*	353151	3.63E-81	9.62E-92–2.63E-65	28

**Reads mapped to *Cryptosporidium parvum* Iowa II**				

*C.parvum*	5807/353152	4.22E-83	9.62E-92–4.12E-39	2608
*C.hominis*	353151	3.52E-75	2.05E-87–3.79E-42	32
*C.ubiquitum*	857276	4.13E-45	8.09E-67–1.35E-23	14

**Reads mapped to *Cryptosporidium hominis***				

*C.parvum*	5807/353152	1.01E-84	9.62E-92–2.52E-44	236
*C.hominis*	353151	1.89E-77	1.17E-90–1.05E-58	20
*C.ubiquitum*	857276	-	4.23E-77–2.02E-74	3

**Reads mapped to *Cryptosporidium meleagridis***				

*C.parvum*	5807/353152	4,19E-83	9.62E-92–2.13E-45	97
*C.hominis*	353151	9,19E-68	2.96E-85–1.34E-62	4
*C.ubiquitum*	857276	-	1.69E-49–1.88E-35	2

**Reads mapped to Apicomplexa**				

*C.parvum*	*5807*	4,19E-83	-	1

*Out of a total of 3,198 confirmed reads, the vast majority (3,095 reads) was confirmed as *Cryptosporidium parvum* by BLASTn, which displayed the highest specificity and thus the lowest median E-value. BLASTn confirmations as *C. parvum* (taxon ID 5807) and *C. parvum* Iowa II (taxon ID 5807) are jointly presented as *C. parvum*.*

## Signature Sequences

To increase specificity and to alleviate computing limitations, metagenomes may be queried using signature sequences, which are specific for the taxon of interest (inclusion ancestor) and are not shared with closely related species (exclusion ancestor). Selection of signature sequences can be achieved by tools such as the high-throughput signature finder (HTSFinder) (Karimi and Hajdu, 2016) and Neptune (Marinier et al., 2017). However, these bioinformatics tools have been developed to identify genomic variation in bacteria. Compared to prokaryotic organisms, parasites possess much larger genomes [42–700 Mb in nematodes, 104–1259 Mb in Platyhelminthes (International Helminth Genomes Consortium, 2019), 23 Mb in the protozoan parasite *Plasmodium falciparum* (Gardner et al., 2002) and 32.8 Mb in *Leishmania* species (Ivens et al., 2005)]. Due to the large genomes of parasites, the above-mentioned signature sequences finding tools cannot be used. To circumvent this limitation, the sequence of individual chromosomes may be used instead of complete genomes to alleviate the size restriction.

We used Neptune to identify signature sequences on chromosome 6 of *C. parvum* Iowa II (inclusion ancestor), using *Cryptosporidium muris* as exclusion ancestor. In total, 365 signature sequences were found, ranging in length between 94 and 5522 nt. However, these signature sequences may not be unique to *C. parvum* when *Cryptosporidium* species other than *C. muris* are considered. Additionally, shorter sequences may be more relevant for metagenome analysis, since metagenomic reads span generally less than 300 nucleotides. To confirm specificity, 247 signature sequences shorter than 500 nucleotides were analyzed with Kraken-2 as taxon classifier. Of these, 187 were correctly classified as *C. parvum* Iowa strain II (see Supplementary File 3). The output of Kraken in our pipeline includes a confidence parameter, ranging from zero to one. Thirty-nine signature sequences were classified with 0.50–1.00 confidence and 148 at lower confidence. Confidence in this case is a measure of how often k-mers have been assigned by Kraken-2 to a given taxon ID. For example, at a confidence value of 0.50, k-mers were assigned to two different taxa IDs: 5806 (*C. parvum*) and 353152 (*C. parvum* Iowa II). While confidence value 0.5 appears very low, it may direct at the same species two times, both *C. parvum* in the example. In another example, a confidence value of 0.30 revealed k-mer assignment to *C. parvum*, *C. parvum* Iowa II and *C. hominis*. Careful evaluation of signature sequence specificity is therefore essential.

**FIGURE 2:**
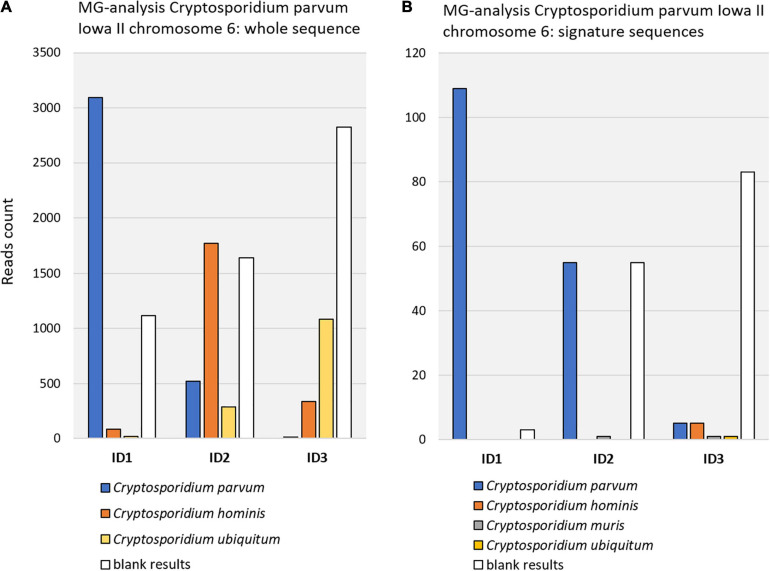
Query results with *Cryptosporidium parvum* Iowa II strain in MG-RAST sample ID 4537108.3 (project calf study digesta, mgp6020). **(A)** Query with chromosome 6 whole sequence. At *C. parvum* strain level, 3095 reads were confirmed (see Table 2 for E-values). **(B)** Query with chromosome 6 signature sequences. Fewer reads were retrieved, due to much shorter reference sequences than in A, but retrieved reads were more specific in comparison to panel **(A)**. Retrieved reads were BLASTn verified. E-values and range ID1: 9E-86 (4E-94–2E-49). E-values and range ID2: 2E-76 (4E-94–2E-06). E-values and range ID3: 3E-70 (4E-94–9E-16). Note that, for improved clarity, the two graphs are not in scale.

Figure 2 shows the result of querying the MG-RAST database project 4537108.3 (calf mid jejunum) using as reference either the *C. parvum* Iowa II whole chromosome 6 or the signature sequences derived from it. Obviously, the latter approach provided much higher specificity at the cost of sensitivity.

## Discussion and Conclusion

The aim of the current study was to provide a proof of principle for the detection of parasites by mining metagenomic public data bases, which may be used for environmental surveillance of these pathogens. Moreover, the specificity of this method was evaluated, based on the idea that reliable identification of parasites in this type of data relies on highly specific results.

The present study provides a proof of principle that parasite DNA can be detected specifically and at high confidence in environmental metagenomic databases. The protozoan *C. parvum* was demonstrated in expected metagenomes (water and small intestinal content from calves) using the whole chromosome 6 sequence as reference sequence. The query results were moderately specific, but in combination with BLASTn, the specificity improved considerably, and the vast majority of reads was confirmed *C. parvum*. This could be further improved when querying the MG-RAST database with specific *C. parvum* signature sequences as reference: only *C. parvum* signals were retrieved at high confidence as shown in the present study. We identified *C. parvum* chromosome 6 (inclusion ancestor) signature sequences in comparison to *C. muris* chromosome 6 (exclusion ancestor). Using these signatures, *C. parvum* was identified in most cases, but also *C. hominis* was retrieved, although at far lower confidence. Chromosome 6 was chosen as a starting point for the proof of principle, since it appeared to generate the most significant results. Due to computing limitations, we could only process one chromosome at a time. However, after computational improvements it is now possible to include much more data and perform signature sequence searches with *C. parvum* whole genomes against 13 genomes of 8 *Cryptosporidium* species other than *C. parvum*, resulting in 75 signature sequences covering all eight chromosomes in one run (data not shown).

From this point on, one way forward would be to identify signature sequences in *C. parvum* compared to *C. hominis* and vice versa, to identify either species specifically in environmental metagenome projects.

Another way to proceed is to query shotgun metagenomic data generated from spiked matrices to evaluate the relevance and to further investigate the limit of detection and the feasibility of quantification of parasites from metagenomic reads.

We used the signature sequence approach to generate species-specific reference sequences to query MG databases, thereby removing redundant parts of the reference DNA, which saves computing power and thus saves time, both during search and post-search analysis. We are now able to run a Kraken2 analysis of metagenome project sequences at 62 Gb per hour, which is roughly 1 Gb per minute. Data storage and searching speed are crucial for MG analysis within reasonable time, but computing time is expected to further decrease with ever expanding computational power in the near future.

In conclusion, querying environmental metagenomic datasets for parasites is feasible and relevant, but requires optimization and validation, similar to the development of new molecular diagnostic assays such as qPCR. Still, this approach could provide access to large numbers of datasets from metagenomic and metatranscriptomic studies. Apart from accessing existing databases, this knowledge will also aid in designing novel dedicated metagenomic projects for detection and typing of parasites in different matrices.

## Future Perspective

A major benefit of exploring public metagenome databases for the presence of parasites is that it covers a range of environments beyond matrices included in most studies, such as soil and (waste)water, which are difficult to investigate using standard parasitological techniques.

Future research should also focus on other foodborne parasites with resistant environmental stages (e.g., *Toxoplasma*, *Giardia*, *Echinococcus*) known to occur in different environments such as soil, surface water, sludge and manure. Isolation methods that effectively extract DNA from such environmental resistant parasite stages must be used.

## Data Availability Statement

The raw data supporting the conclusions of this article will be made available by the authors, without undue reservation.

## Author Contributions

FF designed, performed, and analyzed pipeline runs and wrote the manuscript. IJ supervised the pipeline design and wrote the manuscript. DJ built the pipeline. JG and MP conceived the study and wrote the manuscript. SC and PV helped with the interpretation of the results and wrote the manuscript. All authors contributed to the article and approved the submitted version.

## Conflict of Interest

The authors declare that the research was conducted in the absence of any commercial or financial relationships that could be construed as a potential conflict of interest.

## Footnotes

1 https://www.ncbi.nlm.nih.gov/

2 https://eupathdb.org/eupathdb/

3 https://wormbase.org/

## Supplementary Material

Supplementary Figure 1 Results of querying the MG-Rast metagenome database using the 18S DNA sequence of *Entamoeba gingivalis*. The outer circle shows the species retrieved as a percentage of total retrieved species. The middle circle shows the environment from which species were retrieved. The inner circle shows the percentage of read identity to the *E. gingivalis* query sequence. All sequences showed 100% coverage with the query sequence.

Supplementary Table 1 Panel of selected parasites. Most parasites were found in their expected environments (✓), using BWA-MEM. In some cases, parasites were not found (×).
