# Supplementary material for: Mining Public Metagenomes for Environmental Surveillance of Parasites: A Proof of Principle
Source: Front Microbiol. 2021 Jun 30;12:622356. doi: 10.3389/fmicb.2021.622356 (PMC8278238; doi:10.3389/fmicb.2021.622356)

Supplemental Figure 1. *Entamoeba gingivalis* 18S query in MG-RAST. Forty-three reads with 100% query coverage were found, of which 13 were confirmed with BLASTn (NCBI). At 99 and 98 percent read identity (green and orange wedges), the majority of reads was BLAST-identified as *Entamoeba suis* (environment 'host associated'); at closer inspection, these metagenome reads were retrieved from pig feces. Colors depict numbers of retrieved reads and wedges are shown clockwise in decreasing reads count order, starting with red. The inner circle shows the percentage of identity to the query sequence.

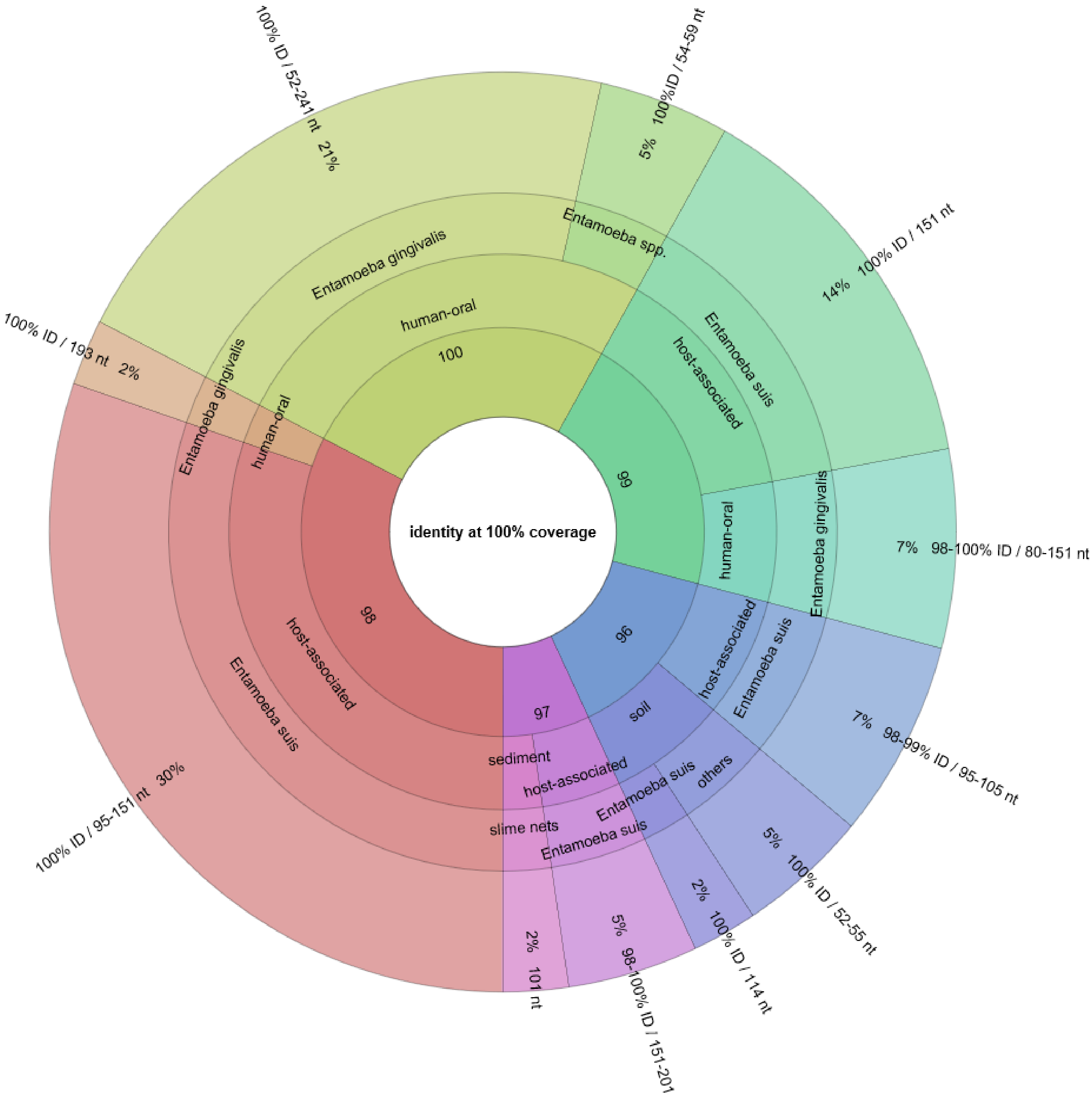

Supplement: Supplementary Figure 1 — Results of querying the MG-Rast metagenome database using the 18S DNA sequence of Entamoeba gingivalis. The outer circle shows the species retrieved as a percentage of total retrieved species. The middle circle shows the environment from which species were retrieved. The inner circle shows the percentage of read identity to the E. gingivalis query sequence. All sequences showed 100% coverage with the query sequence. [file Data_Sheet_1.PDF]
