# Supplementary material for: Mining Public Metagenomes for Environmental Surveillance of Parasites: A Proof of Principle
Source: Front Microbiol. 2021 Jun 30;12:622356. doi: 10.3389/fmicb.2021.622356 (PMC8278238; doi:10.3389/fmicb.2021.622356)
Supplement: Supplementary Table 1 — Panel of selected parasites. Most parasites were found in their expected environments (✓), using BWA-MEM. In some cases, parasites were not found (×). [file Data_Sheet_2.PDF]

**Supplemental Table 1.** Panel of selected parasites; 18S rDNA sequences were used to query the MG-RAST metagenomic database. Most parasites were found in their expected environments (✓), with BWA-MEM alignment. In some cases, parasites were not found (×).

| Parasite | Water | Soil | wastewater sludge | sediment | human oral | host-associated | main hosts |
|---|---|---|---|---|---|---|---|
| *Balantidium coli* | ✓ | ✓ | ✓ | ✓ | | ✓ | Pigs |
| *Cryptosporidium hominis* | ✓ | ✓ | × | ✓ | | ✓ | Humans |
| *Cryptosporidium parvum* | ✓ | ✓ | ✓ | ✓ | | ✓ | Cattle |
| *Entamoeba coli* | ✓ | ✓ | | | | ✓ | Humans |
| *Entamoeba gingivalis* | | | | | ✓ | ✓ | Humans |
| *Trichomonas tenax* | ✓ | | | | ✓ | ✓ | Humans |
| *Trichomonas vaginalis* | | | | | | × | Humans |
| *Tritrichomonas foetus* | | | | | | × | Cattle |
