# Supplementary figures and images for: Mining Public Metagenomes for Environmental Surveillance of Parasites: A Proof of Principle
Source: Front Microbiol. 2021 Jun 30;12:622356. doi: 10.3389/fmicb.2021.622356 (PMC8278238; doi:10.3389/fmicb.2021.622356)

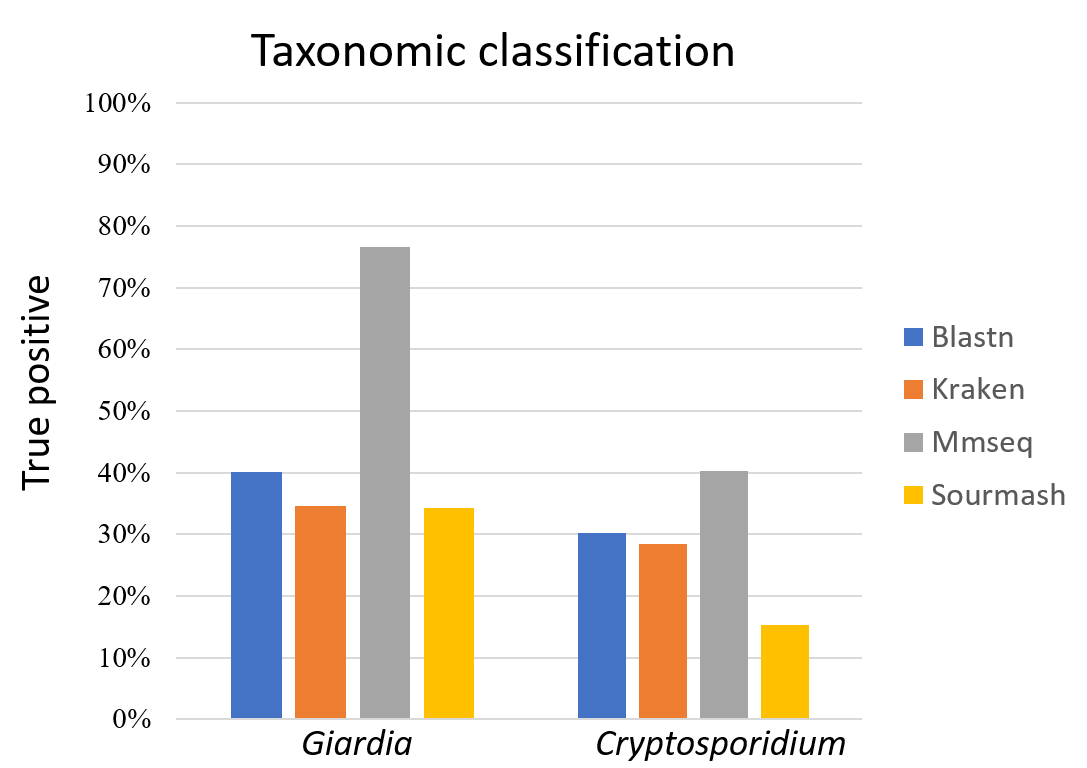

Supplement: Supplementary file 3 [file Image_1.PNG]

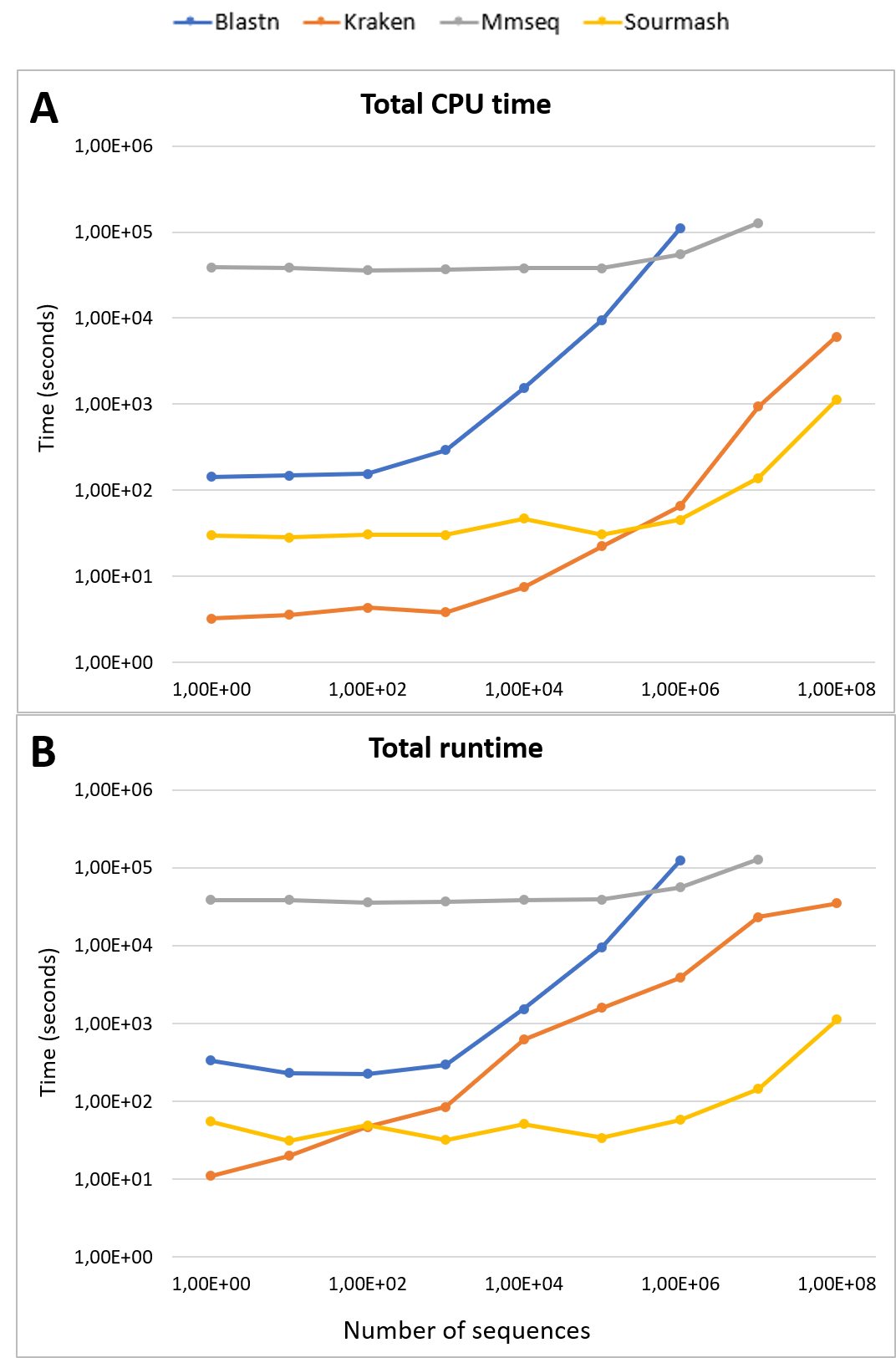

Supplement: Supplementary file 4 [file Image_2.PNG]
