# Supplementary material for: Mining Public Metagenomes for Environmental Surveillance of Parasites: A Proof of Principle
Source: Front Microbiol. 2021 Jun 30;12:622356. doi: 10.3389/fmicb.2021.622356 (PMC8278238; doi:10.3389/fmicb.2021.622356)
Supplement: Supplementary file 5 [file Table_1.DOCX]

Parameter definitions used for pipeline execution as defined in the config file that controls the Python script.

**#Essentials**

RUN_NAME : "xxx" #The name of the run. For example "Bacteria in Soil Kraken".

QUERYDIR : "/path/to/directory" #The path to the directory containing the sub-directories with sequence files. For example "/mnt/db/MG-RAST" will lead to the MG_RAST database that is stored here.

OUTPUTDIR : "/path/to/directory" #The path to the output directory. This is where your output files will be placed.

THREADS : 5 #The amount of threads that are allowed to be used in a core. The maximum amount is 13.

DB : "none" #The name of the external database to use, with the choices being "mg_rast", "none" or "ebi".

**#Pre-processing parameters**

QUALITY_CHECK : "True" #Option that verifies whether to perform a quality check or not. To perform this check, either use "True" or "T" in this option.

META_CHECK : "True" #Option that verifies whether to perform a filter and check based on the meta-data. To perform this check, either use "True" or "T" in this option.

MIN_LENGTH : 75 #The minimum threshold length of a sequence, sequences shorter than this number won't be used in the pipeline.

QUAL_MEAN : 4 #The minimum threshold average quality score of a sequence, sequences of a lesser average quality score than this number won't be used in the pipeline.

MIN_REPLICATES : 2 #The minimum threshold of replicates of a sequence, sequences with more than this amount of replicates won't be used in the pipeline.

MAX_N : 10 #The maximum percentage of unknown (N) bases that a sequence can consist out of. Sequences that hold a number of unknown bases beyond this won't be used in the pipeline.

EOI : "/path/to/file" #The path to the file with the environments of interest, such as 'host-associated', 'water' or 'soil'. Used when only specific environments are of interest, as the other environments won't continue in the pipeline.

COI : "/path/to/file" #The path to the file with the countries of interest, such as 'Canada' or 'United States'. Used when only specific countries are of interest, as the other environments won't continue in the pipeline.

CUSTOM : "/path/to/file" #The path to the file with the custom parameters of choice, such as 'host_taxid 9096', 'seq method Illumina' or 'tissue_material agricultural soil'. Used when only specific parameters are of interest, as the other environments won't continue in the pipeline.

**#Classification parameters**

TOOL : "kraken" #Selection option of the tool to use. Current choices are "kma", "kraken", or "bwa".

THREADS_TOOL : 5 #The threads one instance of the tool is allowed to use. The maximum of threads that can be used is 13.

CONFIDENCE : 0.2 #The minimum accepted confidence score.

**##KRAKEN SPECIFIC**

KRAKENDB : "/mnt/db/kraken2_ntdb/" #The path to the kraken database to be used.

TARGET_GROUP : "D:2" #The taxon and taxid that is used in the kreport file, the letters used in this report (R D K P C O F G S) show the taxonimic levels. R = Root, D = Domain, K = Kingdom, P = Phylum, C = Class, O = Order, F = Family, G = Genus, S = Species. In the kraken script, the taxonomy levels may be chosen.

**##MG_ALIGNER/KMA SPECIFIC**

REFERENCE : "/path/to/reference file" #The path to the reference file used for classification with KMA. When changing the reference file, the current KMA database should be deleted, so a new one can be made.

IDENTITY : 0.05 #The minimum threshold identity of a sequence, sequences with less identity than this number will not be used in the pipeline.

**###MG_ALIGNER**

COVERAGE : 0.8 #minimal coverage threshold

**###KMA**

KMADB: "/mnt/db/KMA_db/" #Local reference database to be used

DEPTH: 2 #this parameter does not change

SCORE : 0.95 #The minimum threshold score for global alignment score of the KMA tool that the results need to meet. Results below this number will not continue in the pipeline.

P_VALUE : 0.05 #The p-value threshold

**#Post-processing parameters**

NONTOOLTHREADS : 10 #The amount of threads that are allowed to be used in a core for the post-processing part of the pipeline. The maximum amount is 13.

SOI : "/path/to/file" #The path to the file containing the tax_ID’s of all species of interest.

**##BLAST SPECIFIC**

DO_BLAST : "False" #Option to choose whether or not to perform BLAST. To use BLAST, either use "True" or "T" in this option.

BLAST_DB : "/path/to/directory/prefix" #The path to the BLAST database directory, this path should end with the prefix for the database of choice, e.g., for the NT-Database use path /mnt/db/NT_Database/nt)

LOCAL : "True" #Indicates whether to perform BLAST locally or remotely. To use BLAST locally, either use "True" or "T" in this option.

EVALUE : 2 #The maximum e-value of a hit in BLAST.

BLAST_IDENTITY : 80 #The minimum identity of a hit in BLAST.

BLAST_COVERAGE : 80 #The minimum coverage of a hit in BLAST.

**#Compare parameters**

NAME_FILE : "/path/to/file" #The file containing the translations from taxid to scientific name of the species of interest.

MIN_HITS : 80

**#Results parameters**

FASTA : "none" #Option to extract the sequences associated with the species of interest. To perform this extraction, either use "True" or "T" in this option.
