# Supplementary material for: Mining Public Metagenomes for Environmental Surveillance of Parasites: A Proof of Principle
Source: Front Microbiol. 2021 Jun 30;12:622356. doi: 10.3389/fmicb.2021.622356 (PMC8278238; doi:10.3389/fmicb.2021.622356)
Supplement: Supplementary file 8 [file Table_7.DOCX]

# Classification evaluation

In the field of data mining, estimation of the quality of used methods is a key step in selecting the most appropriate tool for the problem. Hence the following experiments have been conducted to verify performance and validate significance of the taxonomic classification. Data in these experiments has been collected from the EUpath database (Aurrecoechea, C., et al. (2017)). Both the CryptoDB and the GiardiaDB have been used as query database. Analysis for both parasites were conducted using the inhouse pipeline with Kraken2 (Wood, D. E., Lu, J., & Langmead, B. (2019)), Blastn (Altschul, S. F., et al(1990)), Mmseq (easy-tax) (Mirdita, M., et al. (2020)) and Sourmash (compute and summarize) (Ondov, B. D., et al. (2016)). Blastn and Kraken searched against ncbi’s NT dabase, mmseq has done a six-frame translation search using orf-filter-s 1 against ncbi’s NR database filtered for eukaryotes only, and Sourmash has been searched against the Genbank k=31, 2017.11.07 database provided online. Quality performance has been measured using the whole CryptoDB and GiardiaDB as query database. Time measurements have been created by using Mason 2, simulating 100-bp paired-end illumina sequences from CryptoDB-46_CparvumiowaII_Genome_cleaned_final.fna found in CryptoDB n times. These defaults caused the simulator to simulate sequencing errors at rates of 0.005% for mismatches, 0.005% for insertions, and 0.005% for deletions.

Reliability of the classification step in the pipeline was evaluated by determining the performance of the four taxonomic classification tools mentioned above, against the content of EUpath’s CryptoDB and GiardiaDB. For *Giardia*, correct read identification using Mmseq exceeded the other tools by far (76.68% correctly identified reads) and Blastn yielded 40.10% correctly identified reads; Kraken2 and Sourmash performed similarly, yielding 34.58% and 34.30% correct taxon identification respectively. Using *Cryptosporidium*, similar results were obtained: Mmseq yielded 40.25% correctly identified reads, followed by Blastn (30.13%), Kraken2 (28.40%) and Sourmash (15.30%) (supplemental Figure 3).

# Time complexity

The time complexity analysis was performed on 100-bp paired-end illumina sequences found in CryptoDB (CryptoDB-46_CparvumiowaII_Genome_cleaned_final.fna) simulated with Mason2, similar to the method described by Minimap2: pairwise alignment for nucleotide sequences (Li, H. (2018)). The Initial time difference at 1 sequence could be explained by the different methods used by the classification tools to load query- and target database.

The maximum number of sequences with Blastn and MMseq was was only 10^6^ and 10^7^ respectively, due to the fact that CPU time at these points was already 1000 times higher than with the other two tools.

Kraken displayed a clear difference between CPU time and actual runtime. This is most likely due to a bottleneck other than CPU.

The time to correctly identify query sequences was determined for each of four tools mentioned above, using a range of 1 – 10^8^ sequences per time point. At 1 sequence input, Kraken2 was the fastest tool to obtain results (11 sec), followed by Sourmash (55 sec), Blastn (337 sec) and finaly Mmseq, which took 38975 seconds or 10.83 hours to complete the task.

Looking at CPU time, Blastn and Mmseq intersected between 10^5^ and 10^6^ sequences and from that point on, Mmseq processed input faster than Blastn (supplemental Figure 4A). Also Kraken2 and Sourmash intersected between 10^5^ and 10^6^ sequences and from that point on, Sourmash was faster than Kraken2, but overall, Kraken2 and Sourmash performed similar in CPU time (supplemental Figure 4A), but not in total run time (supplemental Figure 4B).

# Conclusion

From the evaluation of the step it was clear that Mmseq performed classification at the highest precision, but was very slow when not used on a large dataset. For a fast and broad search Sourmash was better suited, but at the cost of precision. Kraken performed at both relatively high speed and precision.

# References

Altschul, S. F., Gish, W., Miller, W., Myers, E. W., & Lipman, D. J. (1990). Basic local alignment search tool. *Journal of Molecular Biology*, *215*(3), 403–410. https://doi.org/10.1016/S0022-2836(05)80360-2

Aurrecoechea, C., Barreto, A., Basenko, E. Y., Brestelli, J., Brunk, B. P., Cade, S., Crouch, K., Doherty, R., Falke, D., Fischer, S., Gajria, B., Harb, O. S., Heiges, M., Hertz-Fowler, C., Hu, S., Iodice, J., Kissinger, J. C., Lawrence, C., Li, W., … Zheng, J. (2017). EuPathDB: The eukaryotic pathogen genomics database resource. *Nucleic Acids Research*, *45*(D1), D581–D591. <https://doi.org/10.1093/nar/gkw1105>

Li, H. (2018). Minimap2: pairwise alignment for nucleotide sequences. *Bioinformatics*, *34*(18), 3094–3100. <https://doi.org/10.1093/bioinformatics/bty191>

Mirdita, M., Steinegger, M., Breitwieser, F., Söding, J., & Karin, L. E. (2020). Fast and sensitive taxonomic assignment to metagenomic contigs. In *bioRxiv* (p. 2020.11.27.401018). bioRxiv. <https://doi.org/10.1101/2020.11.27.401018>

Ondov, B. D., Treangen, T. J., Melsted, P., Mallonee, A. B., Bergman, N. H., Koren, S., & Phillippy, A. M. (2016). Mash: Fast genome and metagenome distance estimation using MinHash. *Genome Biology*, *17*(1), 132. https://doi.org/10.1186/s13059-016-0997-x

Wood, D. E., Lu, J., & Langmead, B. (2019). Improved metagenomic analysis with Kraken 2. *Genome Biology*, *20*(1), 257. <https://doi.org/10.1186/s13059-019-1891-0>

Supplemental Figure captions:

**Supplemental Figure 3: Taxonomic classification.** Four classification tools were included: EUpath's GiardiaDB and CryptoDB using: Blastn (searched against the nt database), Kraken (searched against the nt database), Mmseq (searched against the nr database filtered on eukaryotes) and Sourmash (searched against the K=31, 2017.11.07 database).

**Supplemental Figure 4: Time complexity of different classification tools.** Four classification tools were included: Blastn (searched against the nt database), Kraken (searched against the nt database), Mmseq (searched against the nr database filtered on eukaryotes) and Sourmash (searched against sourmash’s K=31, 2017.11.07 database). Input datasets have been collected using Mason 2, simulating 100-bp paired-end illumina sequences from CryptoDB-46_CparvumiowaII_Genome_cleaned_final.fna found in CryptoDB n times.
